# Increased extent of waterfowl grazing lengthens the recovery time of a colonizing seagrass (*Halophila ovalis*) with implications for seagrass resilience

**DOI:** 10.3389/fpls.2022.947109

**Published:** 2022-08-29

**Authors:** Caitlyn M. O’Dea, Paul S. Lavery, Chanelle L. Webster, Kathryn M. McMahon

**Affiliations:** ^1^Centre for Marine Ecosystems Research, School of Science, Edith Cowan University, Joondalup, WA, Australia; ^2^Department of Water and Environmental Regulation, Aquatic Science Branch, Joondalup, WA, Australia

**Keywords:** recovery time, resilience, grazing simulation, trophic interactions, seagrass, herbivory, Swan-Canning Estuary, swan

## Abstract

Herbivore distributions and abundance are shifting because of climate change, leading to intensified grazing pressure on foundation species such as seagrasses. This, combined with rapidly increasing magnitudes of change in estuarine ecosystems, may affect seagrass resilience. While the overall resilience of seagrasses is generally well-studied, the timeframes of recovery has received comparatively little attention, particularly in temperate estuaries. We investigated how the recovery time (RT) of seagrass is affected by simulated grazing in a southwestern Australian estuary. Whilst excluding swans, we simulated different grazing intensities (25, 50, 75, and 100% removal from 1 m^2^ plots) at four locations in the Swan-Canning Estuary, Western Australia during summer and tracked the recovery of seagrass over 3 months, using seagrass cover as the main measure of recovery. We found that seagrass recovered within 4–6 weeks from the lower grazing intensities (25 and 50%) and 7–19 weeks from the higher grazing intensities (75 and 100%) across the estuary. Increased grazing intensity led to not only longer recovery times (RTs), but also greater variability in the RT among experimental locations. The RT from the higher grazing intensities at one location in particular was more than double other locations. Seagrass recovery was through vegetative mechanisms and not through sexual reproduction. There was a significant grazing treatment effect on seagrass meadow characteristics, particularly belowground biomass which had not recovered 3 months following grazing. As the pressure of climate change on estuarine environments increases, these quantified RTs for seagrass provide a baseline for understanding grazing pressure as a singular disturbance. Future work can now examine how grazing and other potentially interacting pressures in our changing climate could impact seagrass recovery even further.

## Introduction

While trophic interactions between species have evolved over millennia (Durant et al., 2019), they can, nonetheless, fall out of balance as in the case of overgrazing. Overgrazing is a mismatch between the food requirements and food availability in an ecosystem (Durant et al., 2005), and has been observed in aquatic environments through waterfowl (Kollars et al., 2017), sea urchins (Eklöf et al., 2008), and turtle grazing (Fourqurean et al., 2019). This can be the result of increased herbivore abundance and, in extreme cases, has caused the functional extinction of submerged aquatic vegetation (SAV) within an ecosystem (Gangal et al., 2021). In many regions, predicted changes in hydrology have the potential to lead to increased herbivore densities in some aquatic ecosystems, with implications for the plants they depend on. For example, in regions with Mediterranean climates, wetlands can dry, partially or completely, over summer (Brinson and Malvárez, 2002). With a drying climate, these wetlands will become drier for longer periods (Semeniuk and Semeniuk, 2013; Hope et al., 2015) and nearby ecosystems, such as estuaries, are likely to become refuges for waterfowl, potentially increasing herbivore density grazing pressure on SAV.

Seagrasses are a foundation species of SAV in many estuaries (Dayton, 1972) as they create habitat, stabilize sediments, cycle nutrients, and form the base of the trophic web (Hemminga and Duarte, 2000; Unsworth et al., 2015). Like many ecosystems, seagrasses have been under threat from human impact and are declining at alarming rates globally (Waycott et al., 2009; Dunic et al., 2021). Future climate-driven changes in grazer abundance would represent an additional pressure on seagrass ecosystems. The extent of degradation from grazing pressure is often dependent on the abundance and distribution of herbivores (Choney et al., 2014), the scale of impact (O’Brien et al., 2018), and how the plants can respond (Pérez et al., 2012; Sanmartí et al., 2014). Seagrasses are commonly classified into three types based on their ability to resist or recover from disturbance: persistent, opportunistic, or colonizing species (Kilminster et al., 2015). In estuaries, the naturally variable conditions often favor colonizing seagrass species, which are small, fast-growing, and with low resistance to disturbance but the ability to recover rapidly (Kilminster et al., 2015). These seagrasses would be expected to have a relatively high potential for recovery. However, as with recovery following other forms of disturbance, the timeframes of recovery following grazing are not well understood (York et al., 2016).

Understanding recovery time (RT) is particularly important for seagrasses because of the ecosystem services they provide and their vulnerability to ongoing declines (Smith et al., 2016). Knowing how long seagrasses take to recover would allow more accurate prediction of how long they might be vulnerable to additional disturbance, or the length of time required for them, and their ecosystem services (Nowicki et al., 2017; Scott et al., 2021), to return following loss. Limited understanding of RT makes it challenging to determine realistic timeframes for management or intervention (O’Brien et al., 2018), particularly if RT is beyond funding cycles. Greater understanding of RT is likely critical in understanding seagrass resilience and protecting these ecosystems into the future.

Equally important is understanding the mechanism for recovery. Seagrasses are known to recover through three primary mechanisms. Firstly, they expand into unoccupied space by vegetative expansion from surrounding, intact meadows (Marbà and Duarte, 1998). This is common to most seagrasses. Second, they may recover from the germination of seeds in a seedbank. This mechanism seems particularly important in colonizing seagrasses (Kilminster et al., 2015), as these species typically produce large numbers of seeds that accumulate in the sediment. However, little is known about the dormancy of these seeds, their longevity and what breaks dormancy, and consequently it is unclear whether seeds could provide a mechanism of recovery throughout the year or only at certain times. Finally, seagrasses can recover from the immigration of fragments created through breakage of plants in other locations due to physical disturbance (McMahon et al., 2014). Understanding the relative importance of these mechanisms for recovery can guide our understanding of how and how quickly recovery might occur, and the nature of its dependence on adjacent meadows.

Our study investigated if grazing intensity affects the RT of a colonizing species of seagrass. We expected that the RT of seagrass would be slower with increasing grazing intensities; if there is greater removal of seagrass material, there is more area that seagrass needs to replenish to return to the original condition and will subsequently take longer. We also explored which mechanisms explained the patterns in recovery.

## Materials and methods

### Study location and species

**FIGURE 1:**
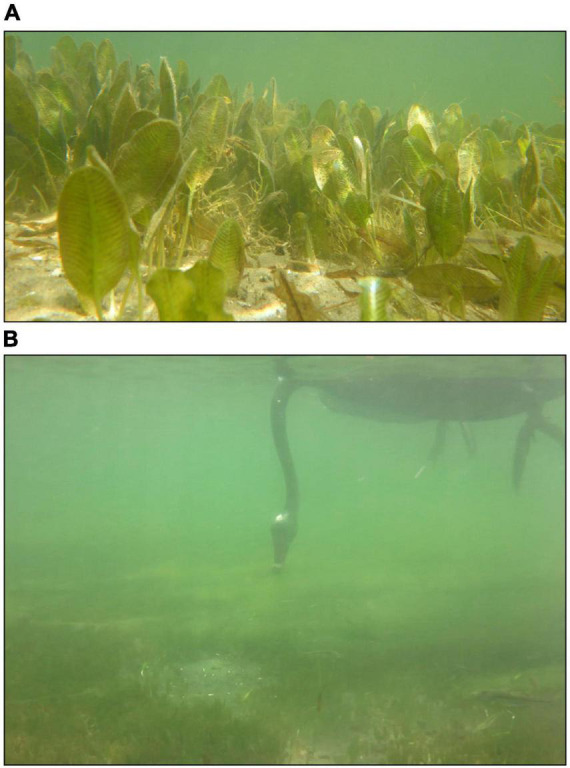
Seagrass (*Halophila ovalis*) in the Swan-Canning Estuary **(A)** and black swan (*Cygnus atratus*) grazing **(B)**.

**FIGURE 2:**
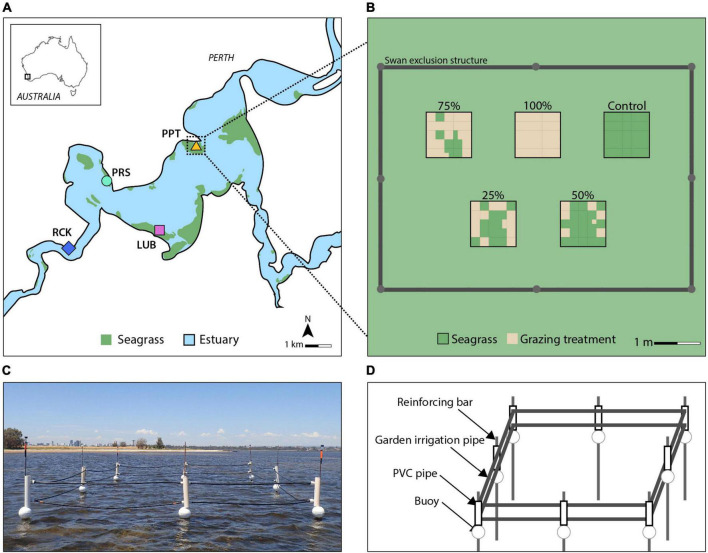
Four replicate blocks (colored symbols) were established across seagrass meadows (green area) in the Swan-Canning Estuary in Western Australia **(A)** (adapted from Forbes and Kilminster, 2014), and conceptual diagram of the replicate block design **(B)**. The grazing treatments show the placement of grazed cells (brown color) which were randomly allocated within a treatment, replicated across the four blocks within seagrass meadows (green color). Swan exclusion structures were constructed to prevent interference at each replicate block **(C)**. Each structure comprised eight steel reinforcing bars (3 m height, 12–16 mm diameter) supported by star pickets (0.5–1 m height) placed at least 1 m into the sediment and in water up to 1 m deep **(D)**. A buoy was threaded along each bar, supporting a two-stranded fence made of garden irrigation pipe threaded through modified PVC pipe brackets separated by a spacer.

A manipulative field experiment was carried out in the lower Swan-Canning Estuary on the Swan Coastal Plain in southwestern Australia (Figure 1). The estuary is permanently open to the ocean and has diurnal oceanic tides with a range of 0.6–0.9 m. Much of the estuary is less than 3 m deep (Thomson et al., 2001). The Swan Coastal Plain also contains transient to permanent wetland areas which provide important habitat for the black swan (*Cygnus atratus* Latham); an iconic herbivorous waterfowl in Australia. Swans are considered “ecosystem engineers” through their impact on the environment which is linked to their behavior, movements, and foraging (Bakker et al., 2016). Swans graze on seagrass meadows in the estuary (Figure 2) and are most abundant during autumn and summer (Storey et al., 1993; Choney et al., 2014). Swans, by virtue of their large size (up to 9 kg), have considerable impacts on their food source, such as seagrass (Wood et al., 2012; Bakker et al., 2016). Swan populations have been shown to consume 25% of daily seagrass production in an Australian estuary (Choney et al., 2014), and up to 20% of annual seagrass biomass in a New Zealand estuary (Dos Santos et al., 2012).

*Halophila ovalis* (R. Br) Hook. F., a colonizing species, is the dominant seagrass in the lower Swan-Canning Estuary (Figures 1, 2). In 2011, *H. ovalis* meadows covered 403 hectares, approximately 16% of the lower estuary (Forbes and Kilminster, 2014). Other species are found in the estuary in mixed meadows with *H. ovalis*, which include *Ruppia megacarpa*, *Zostera muelleri*, *Posidonia australis*, and *Halophila decipiens* (Forbes and Kilminster, 2014). Growth of *H. ovalis* in the estuary is highest during summer and peak biomass is generally reached in December, when temperature, salinity, and light conditions are highest (Hillman et al., 1995), typically followed by peak flowering and fruiting (Kilminster and Forbes, 2014). The experiment was conducted from December 2018 to April 2019, a period of maximum seagrass growth likely to capture the fastest possible RT and maximum swan abundance.

### Grazing simulation experiment

A block experimental design was used to assess the effects of simulated swan grazing on *H. ovalis* seagrass recovery. Four experiment locations (Blocks, 7 × 5 m) were chosen within monospecific meadows of *H. ovalis* in the estuary at Lucky Bay (32°01′16.2″ S, 115°48′47.5″ E), Pelican Point (31°59′16.5″ S, 115°49′28.8″ E), Point Resolution (32°00′06.3″ S, 115°47′26.2″ E), and Rocky Bay (32°01′30.6″ S, 115°46′21.2″ E) (Figure 2). Each block acted as a single replicate location within the estuary. At each location, six levels of simulated grazing were imposed, each in 1 × 1 m experimental plot within the block. As we were not testing for differences among locations, we did not replicate the grazing treatments within each location. Swans were excluded from the experimental blocks to prevent further grazing using exclusion structures. The experiment was established in seagrass meadows with water depths no greater than 1 m and that had no sign of recent grazing activity. Within each block, the placement of grazing treatment plots was randomly configured. The six treatments were (1) Control: no simulated grazing used as a reference to determine the RT of the grazed plots; (2) 25%: removal of 25% of total seagrass biomass from the 1 m^2^ as simulated grazing; (3) 50%: removal of 50%; (4) 75%: removal of 75%; (5) 100%: removal of 100%; and (6) Procedural Control (PControl): a 1 m^2^ plot marked in the surrounding meadow at least 1 m from the boundary of the block without grazing exclusion and used to test for experimental artifacts in comparison to Control plots inside the structures. Individual swans can devegetate areas with diameters between 0.3 and 1.5 m, with an average size of 0.28 m^2^ (Dos Santos et al., 2012), represented by the 25% treatment in this experiment and treatments with greater removal represented increased grazing intensity. The seagrass cover in the PControl plots was not different to that in the Control plots over the duration of the experiment (Supplementary Figure 1 and Supplementary Table 2), indicating that the exclusion structures did not impact the cover of seagrass.

The temporary exclusion structures were placed around each replicate “block” (experimental and Control plots, but not the PControl) to prevent natural grazing activity (Figure 2). The exclusion design was adapted from a similar experiment conducted in Chesapeake Bay United States to exclude mute swans (*Cygnus olor*, Gmelin) (Tatu et al., 2007) and which had no negative impacts on swans or other fauna (J. Anderson, personal communication). The structure used in the present study was designed to minimize interference with light availability and water flow. Birds were observed occasionally perching and/or roosting on the structures. The structure always remained above the surface of the water to prevent swan access throughout the tidal regime, while ensuring swan welfare. This study was reviewed and approved by the ethics committee at Edith Cowan University (project 21327).

Grazing was simulated on January 17, 2019 by dividing each 1 m^2^ treatment plot into 25 equally sized cells (0.04 m^2^) and removing seagrass biomass within a random selection of cells from each plot according to the treatment (Figure 2). Seagrass was removed using similar methods employed by Eklöf et al. (2009) and Choney (2012): rhizomes were cut with a core (∅ 9.6 cm) which encompassed most of one cell, and the plant material removed by hand. The seagrass was sieved so that most of the sediment was placed back into the cell.

### Data collection and analysis

#### Swan presence

The number of swans within line of sight was assessed visually was recorded at each block and at each sampling time. There was no evidence of natural grazing scars within or near the experiment, except at one block Rocky Bay (RCK) at the end of the experiment period. Due to this interference, data is unavailable for biomass characteristics and recovery mechanisms in the 25% grazing treatment at RCK.

#### Percent cover

*Halophila ovalis* cover (%) was measured within each treatment, Control and PControl plot to determine the RT following simulated grazing treatments. Cover was recorded before grazing was simulated (time = 0), immediately following grazing, and then 14, 25, 49, 63, and 77 days following grazing. To measure cover, all 25 cells within each plot were photographed. A single image was taken of each cell and analyzed using SeaGIS TransectMeasure software. A regular grid of 25 measuring points was overlaid on each image and the presence/absence of seagrass at each point was recorded at each point. The points with seagrass present were pooled across all 25 images taken for one plot, and was divided by the total number of points across the plot (625 points measured over 1 m^2^ plot) to provide an overall cover (%). Due to low tides which prevented photography, data are unavailable for all treatments on Day 0 at Lucky Bay (LUB) and for 25% at RCK. On rare occasions, treatments were unable to be sampled due to equipment failure: the PControl on Day 49, and 75% on Day 49 and 77 at Pelican Point (PPT); and the 25% on Day 49 at Point Resolution (PRS). Five to seven weeks following simulated grazing, a substantial proportion of *H. ovalis* leaves were shed from the plants as part of regular senescence resulting from reducing temperatures (Hillman et al., 1995). The PControl also followed this trend, indicating the reduction in cover was unlikely a result of the experiment treatments (Supplementary Figure 1). The natural grazing that occurred at RCK at the end of the experiment occurred at 77 days and therefore did not affect the collection of this data.

#### Recovery time

The RT of seagrass in each treatment plot was defined as the number of days required for the cover (%) in a treatment plot to reach the minimum cover observed in the Control plot over the experimental period. Thus, the cover criterion for recovery was set independently at each block, as there was variation in seagrass cover in the Control plots among blocks. RT for each treatment was calculated by plotting the change in cover over time, fitting a curve to this relationship and then using this curve to estimate the earliest time at which the cover of a treatment intercepted the minimum cover of the Control plot at the same block (Supplementary Figure 2). In some cases, the cover of seagrass in the treatments did not reach the minimum cover observed in the corresponding Control during the experiment, so the curve was extrapolated beyond the time of the experiment to determine the RT. For many plants, growth in the early life stages often follows an exponential trajectory, as has been demonstrated for the cumulative rhizome length of *H. ovalis* which exhibits linear growth in initial stages then exponential (Marbà and Duarte, 1998). Consequently, an exponential curve was applied to the data to describe the change in cover over time:


$$\text{y}\,a\,e^{b\,x}$$

where *y* = cover (%) at a given time, *a* = cover (%) at Day 0 following simulated grazing, *e* = Euler’s Number (∼2.7182), *b* = growth constant or continuous rate of increase in cover, and *x* = time (days).

#### Biomass characteristics

The biomass characteristics (leaf density, aboveground biomass and belowground biomass) were assessed 84 days following simulated grazing by destructively sampling within each plot. Three replicate samples were collected using cylindrical cores (9.6 cm diameter × 15 cm depth) in the same three randomly selected cells from each plot. The collected material was placed in calico bags and transported to the laboratory and stored at −5°C until processed. In the laboratory, the plant material was rewashed in estuary water, and the leaves scraped to remove excess sediment and epiphytic material. The leaves and petioles were separated from the rhizomes using a blade, and the leaf density, aboveground (leaf + petiole) and belowground (rhizome + root) biomass (g DW, following drying at 60°C) was recorded per 0.007 m^2^. Data are unavailable for RCK in the 25% treatment due to natural grazing occurring before sample collection.

#### Recovery mechanisms

The experimental sites were monitored on day 14 and day 25, to determine whether the mechanism of recruitment of new vegetative material into grazed areas was through: (a) rhizome extension from surrounding seagrass patches, characterized by unbroken rhizomes extending into “grazed” areas from the adjacent meadow; (b) establishment of vegetative fragments, where there was no rhizome connection between the recruited material and the surrounding meadow; or (c) germination of seeds. The presence of each recovery mechanism was recorded for all “grazed” cells within each treatment. Due to the nature of the simulated grazing treatments, with different treatments having a different number of grazed cells, the data were pooled across the two sampling periods and expressed as a proportion of the number of cells measured in each plot at each location.

The samples collected for the biomass were further processed to determine the characteristics related to potential mechanisms of recovery. These were: (a) node density, (b) the ratio between above and belowground biomass which can indicate the allocation of resources, (c) branching frequency (number of formed branches, as a proportion of node density), and (d) branching potential frequency (number of apical buds, as a proportion of node density) which can all indicate the patterns of clonal growth; (e) flowering and fruiting frequency (as a proportion of nodes), which can indicate potential investment in sexual reproduction.

#### Statistical analysis

To test for differences in RT, the data were analyzed using a permutation two-way analysis of variance (using Primer v6+ and PRIMER-E) with two factors: (1) Treatment: 25, 50, 75, and 100% grazing nested in Block; and (2) Block: LUB, PPT, PRS, and RCK. The PERMANOVA analysis was run on the resemblance matrix using Euclidean distance (*p*-value = 0.05) with between 822 and 840 unique permutations for each treatment combination. The dispersion of the data was tested on raw data using PERMDISP (*p*-value = 0.05). The null hypothesis for the analysis was there was no difference in RT across the four grazing intensity treatments. Permutation pair-wise tests were performed, following significant mains-test, to determine which levels within each factor were significantly different. A PERMANOVA was conducted on the meadow characteristics and recovery mechanisms to test for significant changes in each variable for Treatment, Block, and interactions (Treatment × Block) as fixed factors. Main and pair-wise tests were run as described for RT.

To test if the aboveground cover (%) of seagrass could predict the characteristics of the meadow (leaf density, aboveground biomass and belowground biomass), we pooled all replicate blocks and treatments and calculated Spearman rank correlation coefficient (ρ) with a *p*-value of 0.05. This was done for each of the three meadow characteristics in each treatment with the seagrass cover at the corresponding time point (Supplementary Figure 3).

## Results

### Swan presence

Swans were observed at all blocks except PRS, at least once during the experiment (Supplementary Table 3). Swans were observed the most at LUB on almost 90% of sampling occasions and at PPT more than 60% of the time, with up to 60 and 35 individuals observed at one time, respectively. At RCK, two individuals were observed grazing immediately beside the exclusion structure, but only once (63 days following simulated grazing). Natural grazing was observed in the 25% treatment at RCK on the following sampling occasion.

### Cover

**FIGURE 3:**
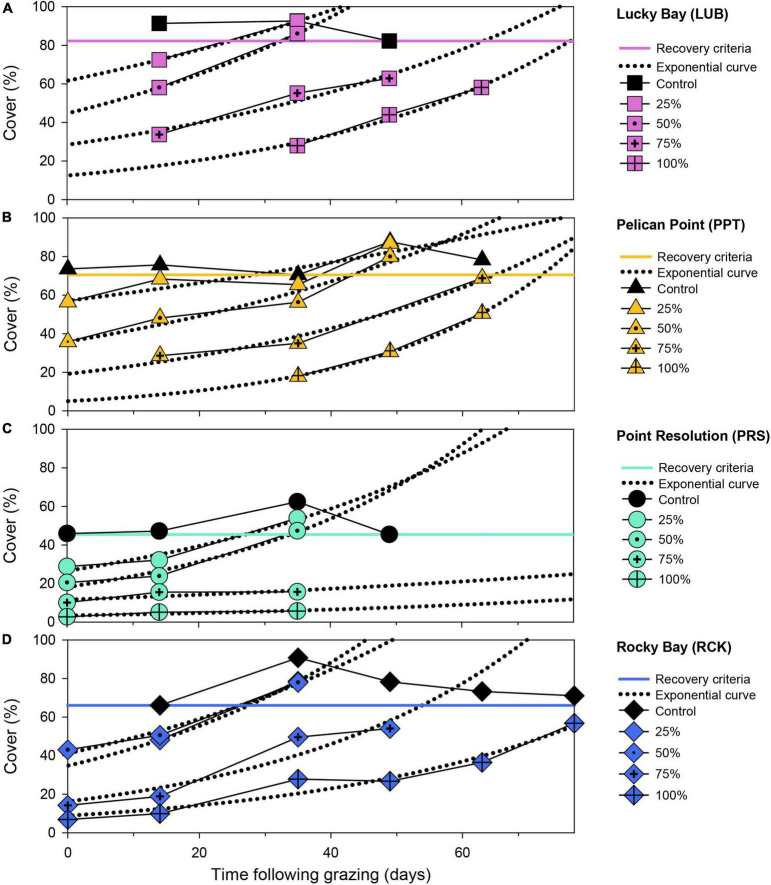
Seagrass aboveground cover (%) over time following simulated grazing (simulated on Day 0) at four replicate block locations: Lucky Bay **(A)**, Pelican Point **(B)**, Point Resolution **(C)**, and Rocky Bay **(D)**. Data is the cover of each treatment plot at each replicate block location (solid lines with colored symbols) with an exponential curve applied (dotted black lines). The minimum recovery time (RT) for seagrass cover was determined at the point at which the fitted curve intercepted the minimum cover observed in the control plot (solid colored line). Missing points is where data is unavailable.

Seagrass cover in the control plots was variable over the experiment but was consistently higher than in the treatments, and the cover decreased toward the end of the experiment due to senescence (Supplementary Figure 1). The grazing simulation effectively reduced seagrass cover in each treatment and was proportional to the treatment imposed. Cover increased in all treatment plots over the experiment, but the extent of these increases varied among treatments. By the end of the experiment (77 days post-grazing), 13 of the 16 treatment plots were more than 70% of the respective control plots and RTs could be calculated within this timeframe. However, some treatments that did not reach the criteria and the RT was extrapolated (Figure 3).

### Recovery time

**FIGURE 4:**
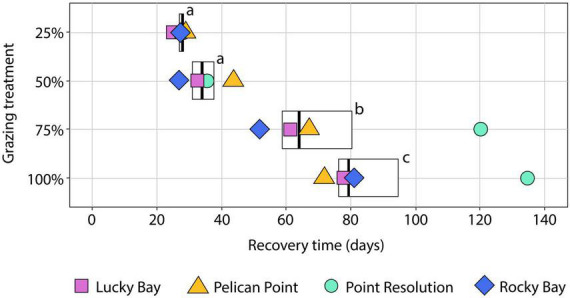
Boxplot of the Recovery Time (RT) in days of *Halophila ovalis* based on cover (%) following different intensities of simulated grazing (removal of 25, 50, 75, and 100%). Lower and upper box boundaries are the 25th and 75th percentiles, respectively, the dark vertical line is the median, the lower and upper error bars are the 10th and 90th percentiles, respectively. The symbols indicate the RT at the replicate blocks in the Swan-Canning Estuary: Lucky Bay (LUB), Pelican Point (PPT), Point Resolution (PRS), and Rocky Bay (RCK). Letters indicate statistical significance among RT for treatments based on permutational pair-wise *post-hoc* analysis (Supplementary Table 4, *p* ≤ 0.05). Where bars share the same letter, there is no significant difference.

The RT following simulated grazing ranged from 25 to 135 d and, generally, increased with the intensity of grazing (Figure 4). PERMANOVA indicated a significant effect of treatment on RT (*F* = 12.489, *p* < 0.05, Supplementary Table 1). *Post-hoc* analysis showed no significant difference in the mean RT between the 25 and 50% grazing treatments (Supplementary Table 4, *p* > 0.05, Figure 4), which averaged 28 ± 1 and 35 ± 3 d, respectively. The 75% grazing treatment had a significantly (Supplementary Table 4, *p* < 0.05, Figure 4) longer RT (75 ± 13 d), and the longest RT was the 100% treatment (92 ± 13 d) which was significantly different to all other treatments (Supplementary Table 4, *p* < 0.05, Figure 4). The variability in RT also increased with grazing intensity, with a small coefficient of variation (CV) in the 25% treatment of 6% increasing to 21% in the 50% treatment, to 41% in the 75% treatment and 32% in the 100% treatment. This increase in variability was due, primarily, to the much longer RT for the 75 and 100% grazing treatments at the PRS block, which were nearly two times longer than in the other blocks.

### Biomass characteristics

**FIGURE 5:**
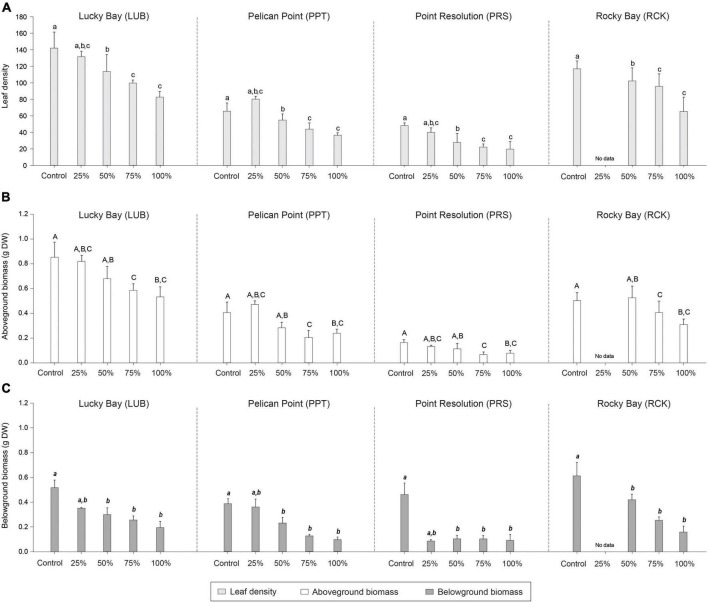
Mean leaf density (**A**; light gray bars), aboveground biomass (**B**; white bars), and belowground biomass (**C**; dark gray bars) in the grazing treatment plots (25, 50, 75, and 100% removal) and Control plots at the four replicate block locations across the Swan-Canning Estuary: Lucky Bay (LUB), Pelican Point (PPT), Point Resolution (PRS), and Rocky Bay (RCK). Statistical significance (pairwise test) is indicated in lowercase letters for leaf density, uppercase letters for aboveground biomass, and bold italic letters for belowground biomass based on pairwise *post-hoc* comparison summarized in Supplementary Table 5 (*p* ≤ 0.05). Where bars share the same letter, there is no significant difference. Pairwise testing indicated significant differences among blocks for leaf density (LUB = RCK > PPT > PRS), aboveground biomass (LUB > RCK > PPT > PRS) and belowground biomass (LUB = RCK > PPT > PRS) (Supplementary Table 5; *p* ≤ 0.05). Data are the means (*n* = 3) ± standard error.

PERMANOVA identified significant differences among treatments in the leaf density (*F* = 10.819, *p* < 0.05), aboveground biomass (*F* = 6.473, *p* < 0.05) and belowground biomass (*F* = 15.996, *p* < 0.05), 84 days after simulated grazing (Supplementary Table 1). *Post-hoc* analysis indicated there were no statistically significant differences between the Control and PControl plots for these characteristics (Supplementary Table 5, *p* > 0.05), but there were among the Control plots and the higher grazing treatments (Supplementary Table 5, *p* < 0.05, Figure 5). Although there were significant differences among blocks, as the interaction term was not significant, the response of the grazing treatments was consistent among blocks.

By 84 days following simulated grazing, the leaf density and belowground biomass was the same as the Control only in the 25% grazing treatment (Table 1 and Figure 5). For aboveground biomass, there was no difference between the Control and the 25 and 50% grazing treatments (Table 1, Figure 5, and Supplementary Table 5), but all other treatments were lower than the controls, despite the observed or predicted recovery of seagrass cover for all treatments (except for the high grazing treatments at PRS) by that time (Table 1). The exceptions to this were the 75 and 100% grazing treatments at the PRS block, where recovery based on cover had also not occurred by 84 days.

**TABLE 1:** Comparison between the minimum recovery time (**RT**) (days) based on aboveground cover (%) to the meadow characteristics sampled 84 days post-grazing treatments.

Grazing treatment	Recovery time (days)				Leaf density or belowground biomass				Aboveground biomass			
			
	LUB	PPT	PRS	RCK	LUB	PPT	PRS	RCK	LUB	PPT	PRS	RCK
25%	25	29	28	28	✓	✓	✓	✓	✓	✓	✓	✓
50%	33	44	35	27	x	x	x	x	✓	✓	✓	✓
75%	61	67	120	52	x	x	x	x	x	x	x	x
100%	78	72	135	81	x	x	x	x	x	x	x	x

Cell color indicates if the treatment was predicted to have recovered at the time of sample collection (blue = recovered, red = not recovered) and the symbols indicate if the meadow characteristic was recovered based on the difference to the Control plot (tick = characteristic not significantly different to Control, cross = significantly different to Control).

For pooled data across all replicate blocks and treatments, the Spearman’s rho correlation analysis indicated a significant and strong positive correlation between the leaf density and cover (*r* = 0.71, *p* < 0.001), and between aboveground biomass and cover (*r* = 0.71, *p* < 0.001), and significant, moderately positive correlation between the belowground biomass and cover (*r* = 0.41, *p* < 0.001; Supplementary Figure 3).

### Recovery mechanisms

The 25, 50, and 75% grazing treatments at all replicate blocks had recruitment of *H. ovalis* into all the cleared cells *via* rhizome extension in the first 4 weeks following simulated grazing. There was rhizome extension observed in 70–100% of the cells in the 100% treatments (Table 2). Recruitment from the settlement of vegetative fragments was observed in 40–46% of cells in the higher intensity grazing treatments (75 and 100%), and less in the lower grazing treatments (12–19% of cells). No recruitment from seedlings was observed throughout the experiment. Visual inspection in the field and in the cover photographs indicated that grazed cells near the edge of the plot regenerated before grazed cells in the center of the plot.

**TABLE 2:** Proportion (%) of recovery due to (1) rhizome extension and (2) vegetative fragments observed up to 4 weeks following grazing simulation (25, 50, 75, and 100% removal), as a proportion (%) of the number of grazed cells measured in each treatment, per replicate block in the Swan-Canning Estuary: Lucky Bay (LUB), Rocky Bay (RCK), Pelican Point (PPT), and Point Resolution (PRS).

Block	Treatment			
	
	25%	50%	75%	100%
**(1) Rhizome extension**				
LUB	100	100	100	100
RCK	100	100	100	90
PPT	100	100	100	90
PRS	100	100	100	70
Mean	100 ± 0	100 ± 0	100 ± 0	87.5 ± 6.3
**(2) Vegetative fragments**				
LUB	0	25	66.7	70
RCK	50	50	66.7	40
PPT	0	0	16.7	30
PRS	0	0	33.3	20
Mean	12.5 ± 12.5	18.75 ± 12.0	45.8 ± 12.5	40 ± 10.8

**FIGURE 6:**
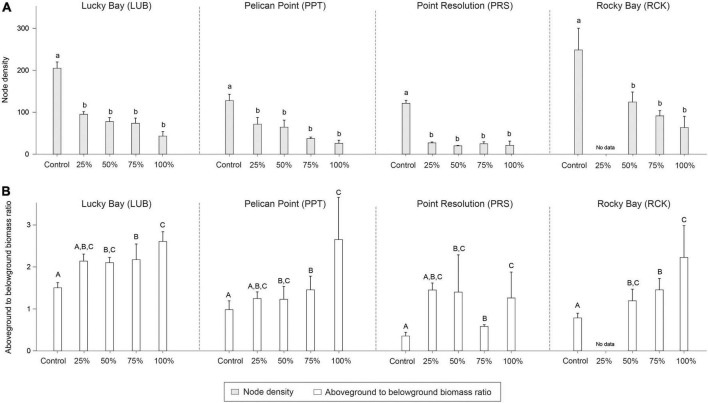
Mean node density (**A**; light gray bars) and ratio of aboveground to belowground biomass (g DW) ratio (**B**; white bars) in the grazing treatment plots (25, 50, 75, and 100% removal) and Control plots at the four replicate block locations across the Swan-Canning Estuary: Lucky Bay (LUB), Pelican Point (PPT), Point Resolution (PRS), and Rocky Bay (RCK). Statistical significance (pairwise test) is indicated in lowercase letters for node density and uppercase letters for the aboveground to belowground biomass (g DW) ratio based on pairwise *post-hoc* comparison summarized in Supplementary Table 6 (*p* ≤ 0.05). Where bars share the same letter, there is no significant difference. Pairwise test indicated significant differences among blocks for node density (RCK > LUB > PPT > PRS) and aboveground to belowground biomass (g DW) ratio (LUB > RCK = PPT > PRS) (Supplementary Table 6; *p* ≤ 0.05). Data are the means (*n* = 3) ± standard error.

**FIGURE 7:**
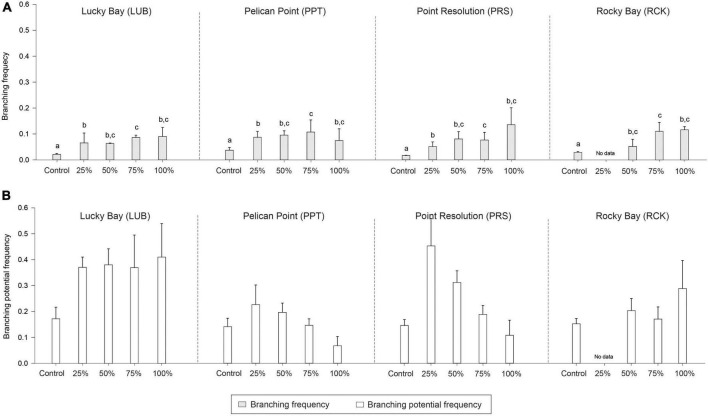
Mean branching frequency (**A**; light gray bars) and branching potential frequency (**B**; white bars) in the grazing treatment plots (25, 50, 75, and 100% removal) and Control plots at the four replicate block locations across the Swan-Canning Estuary: Lucky Bay (LUB), Pelican Point (PPT), Point Resolution (PRS), and Rocky Bay (RCK). Statistical significance (pairwise test) is indicated in lowercase letters for branching frequency based on pairwise *post-hoc* comparison summarized in Supplementary Table 6 (*p* ≤ 0.05). Where bars share the same letter, there is no significant difference. If the main PERMANOVA test showed no significant effect, then no pairwise results are included. Pairwise testing indicated significant differences among blocks for branching frequency (LUB > PRS > PPT, LUB > RCK) (Supplementary Table 6; *p* ≤ 0.05). There was no statistical significance for branching meristem frequency (*p* > 0.05). Data are the means (*n* = 3) ± standard error.

**FIGURE 8:**
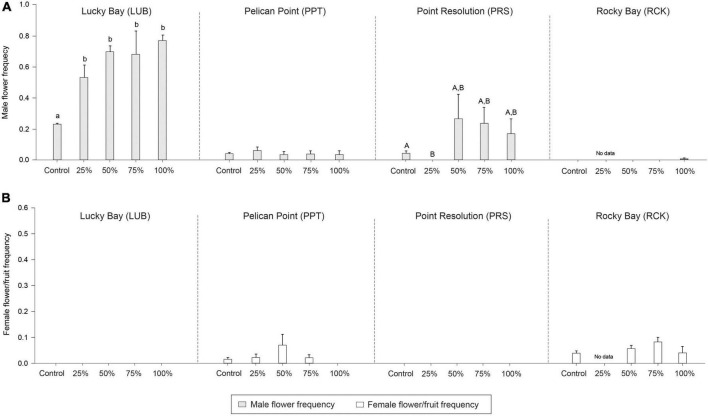
Mean male (**A**; light gray bars) and female (**B**; white bars) reproduction frequency in the grazing treatment plots (25, 50, 75, and 100% removal) and Control plots at the four replicate block locations across the Swan-Canning Estuary: Lucky Bay (LUB), Pelican Point (PPT), Point Resolution (PRS), and Rocky Bay (RCK). Statistical significance (pairwise test) is indicated based on pairwise *post-hoc* comparison summarized in Supplementary Table 7 (*p* ≤ 0.05). Pairwise testing also indicated a significant interaction between treatment and block for male reproduction frequency (Supplementary Table 7; *p* < 0.05). Where bars share the same letter, there is no significant difference. If the main PERMANOVA test showed no significant effect, then no pairwise results are included. There was no statistical significance for female reproduction frequency (*p* > 0.05). Data are means (*n* = 3) ± standard error.

There were significant differences in node density among treatments (PERMANOVA; *F* = 31.441, *p* < 0.05), branching frequency (*F* = 11.941, *p* < 0.05), and above to belowground biomass ratio (*F* = 9.693, *p* < 0.05) between the treatment and control plots, and sometimes there were also differences among blocks (Supplementary Table 1). While *post-hoc* analysis indicated there were significantly less nodes in all treatments compared to the Control plots (Supplementary Table 6, *p* < 0.05, Figure 6), there was significantly more branching in the grazing treatments (Supplementary Table 6, *p* < 0.05, Figure 7). The aboveground to belowground biomass ratio was significantly higher in grazed plots than in Controls (Supplementary Table 6, *p* < 0.05, Figure 6), and this effect generally increased with the intensity of grazing. There were no significant differences observed between the Control plots and treatments for branching potential frequency (Supplementary Table 6, *p* > 0.05, Figure 7). There was a significant interaction between Block and Treatment observed for the frequency of male flowers (Supplementary Table 1, *F* = 2.962, *p* < 0.05), with *post-hoc* analysis showing a greater proportion of male flowers observed in treatments compared to the control but at LUB only (Supplementary Table 7, *p* < 0.05, Figure 8). There were also significant differences identified at PRS, but in this case it was due to the absence of flowers in the 25% treatments. There were no differences for female flower frequency for any factor (Supplementary Table 1, *p* > 0.05, Figure 8).

## Discussion

The findings supported our hypothesis that greater extent of grazing pressure resulted in slower RT of seagrass cover, with higher intensity grazing requiring between 2.5 and 5 times longer (11–20 weeks) to achieve recovery than lower intensity grazing (4–5 weeks). These findings are broadly consistent with O’Brien et al.’s (2018) generalization that recovery is influenced by the spatial extent of disturbance, with recovery delayed or compromised when a larger area is impacted. These recovery times (RTs) were even greater for other seagrass attributes, such as below-ground biomass. Not only did the RT increase with greater grazing pressure, but so too did the variability in the time required for recovery. Somewhat unexpectedly, there was no recovery from seed germination, instead it was overwhelmingly due to vegetative growth from the surrounding meadow with some contribution from settlement and growth of seagrass fragments. We discuss these findings in context of seagrass resilience and consider their implications for estuarine seagrasses under changing climate regimes.

Grazing is a physical and mechanical disturbance that removes biomass (Valentine and Heck, 1999), and in this study, the initial impact to seagrass cover was proportional to the treatment intensities simulated. As recovery of the disturbed patch requires either vegetative expansion from the surrounding meadow, immigration of fragments parts of adult plants, or the germination of seeds stored in seed banks or immigrated from other areas, when more biomass is removed it takes longer to recover. If recovery mechanisms were driven by immigration, this could reduce the RT, as the immigrants would more likely fill in the space in a random manner, whereas if recovery was driven by growth from the remaining meadow, the timescale of recovery would be longer with a greater extent of disturbance, as it is strongly dependent on the size and spatial orientation of empty patches and in-filling from the edge of disturbance (Rasheed, 2004; Rasheed et al., 2014). The plants in this study relied entirely on vegetative mechanisms to recover, mostly through the expansion of rhizomes from the surrounding ungrazed meadow, which explains the dependence of RT on disturbance intensity. A small proportion of recruitment was from fragments, a novel finding for this species (McMahon et al., 2014), and no recovery from seed. While recovery through sexual reproduction could have been expected for this colonizing species of seagrass (Kilminster et al., 2015), recovery from seed was not observed in this study. While *H. ovalis* can develop large seed bank reserves and this has been documented locally (Kilminster et al., 2015; Webster et al., 2021), germination is most likely to occur in spring (Statton et al., 2017), a period not captured in this study. The time of year when recruitment from seed germination is likely to occur coincides with periods of slower plant growth (Ronald et al., 2014; Cubas, 2020), whereas this study was conducted in summer during the peak growth period for this seagrass (Hillman et al., 1995; Kilminster and Forbes, 2014). The findings indicate that even in a colonizing species of seagrass, regrowth from surrounding meadows may be integral to the recovery, especially during periods when recruitment from seed does not occur and, consequently, recovery could be delayed or prevented in less dense or patchy meadows or following large-scale or repeat disturbance (Preen, 1995; Gangal et al., 2021). If, indeed, the dependence of recovery on vegetative regrowth is a result of the timing of the experiment, and because growth rates are highest at this time of year, then the RTs we report here are likely to be faster than at other times of year when growth rates are slower. However, this assumes that rhizome extension is the primary source of recovery at all times, which is unlikely given that *H. ovalis* produces viable seedbanks (Kuo and Kirkman, 1992). It is possible that at some times of year, the seedbank may provide an alternative source of recovery, though it is not possible from our results to predict how enhanced seed-based recovery might also influence the timeframe of recovery. It is also not completely clear whether recovery from seeds could occur throughout the year, or only at some times. *H. ovalis* seeds are known to have a dormancy period and so release from that dormancy would be required for seed-based recovery (Kuo and Kirkman, 1992). For some seeds, physical disturbance (such as that caused by grazing) is known the break the dormancy of seeds (Peterken and Conacher, 1997), but our findings suggest that this is not the case here. This is consistent with finding of Statton et al. (2017), that *H. ovalis* seeds require changes in temperature to break the dormancy. Thus, we conclude that recovery occurs through vegetative expansion, without excluding the possibility for other mechanisms at other times of year.

Rapid clonal growth and in-filling of patches is facilitated by branching (Marbà and Duarte, 1998; Kilminster et al., 2015). This was evident with 1.8–8.2 times more branching in the recovered grazed patches compared to ungrazed seagrass. This increased formation of branches to facilitate recovery has been recorded for this species following dugong grazing (Preen, 1995; Nakaoka and Aioi, 1999) and other mechanical disturbances like boat anchor scarring (Widmer, 2006). These studies also found similar recovery timeframes of weeks to months. In contrast, RT for other, persistent or opportunistic seagrass species is much longer for disturbance of similar spatial scales. For *Z. muelleri* meadows following simulated swan grazing (Dos Santos et al., 2013), *Thalassia*, *Syringodium*, and *Posidonia* meadows following urchin grazing (Eklöf et al., 2008), and in *Posidonia* meadows following removal from boat moorings (Glasby and West, 2018), the RT is in the order of years to decades. This suggests that recovery timescales are dependent on the species and life history strategy (Kilminster et al., 2015), and rapid recovery cannot be assumed for all seagrass meadows. The species or life history should be an important consideration when determining the timeframe of how monitoring programs set recovery targets. This is particularly important as the condition or persistence of meadows can be influenced by external factors over space and time (O’Brien et al., 2018).

Two possible factors could explain the greater variability in the RTs following higher grazing intensity: (1) disruption of self-sustaining feedbacks following disturbance at larger scales; or (2) population-specific resilience to grazing. When loss occurs at a larger scale, recovery can become delayed and difficult to predict (O’Brien et al., 2018). In the first case, the RT at one location in the Swan-Canning Estuary was twice as long as other areas, and at this location there is greater exposure to prevailing south-westerly winds and closer proximity to boat traffic. These features could result in hydrodynamic conditions that resuspend sediment, particularly where seagrass is removed, resulting in rhizome disturbance and light limitation that hinders seagrass growth and recovery (Nowicki et al., 2017; O’Brien et al., 2018), a well-known feedback system in seagrass ecosystems (Ralph et al., 2007; Reise and Kohlus, 2008; Adams et al., 2016; Maxwell et al., 2017). Alternatively, the distribution of herbivores across many aquatic and terrestrial ecosystems can be concentrated to areas with preferable forage or habitat conditions (Lefévre and Bellwood, 2011; Owen-Smith, 2014). Dense herbivore populations can intensify grazing activity to certain areas within an ecosystem, affecting the consumption and distribution of plant communities (Lefévre and Bellwood, 2011). Seagrass meadows that host dense herbivore populations could develop population-specific resilience to grazing (White et al., 2011). Terrestrial plant populations with previous exposure to herbivory can develop higher tolerance than populations with low levels of herbivory (Boalt et al., 2010). The seagrass meadows in this study with the fastest RTs have been recorded as swan population “hotspots” (Choney et al., 2014), which suggests these meadows may be more resilient to grazing than others, in the same way that population-specific resilience of seagrass in this system has been observed following exposure to hyposalinity (Webster et al., 2021) and is being actively explored in seagrass ecosystems more generally (Bennett et al., 2021). Irrespective of the cause, the greater variability and reduced predictability of recovery response to higher magnitude of disturbance highlights that RT can be variable at the local scale (Smith et al., 2016). Understanding the existing grazing pressure, local environmental conditions, and recovery mechanisms will help to identify if a seagrass ecosystem is vulnerable (Jenkins et al., 2015), and if it could be appropriate for “future-proofing” restoration efforts (Wood et al., 2019). The results of this study should, however, be taken in context of the small-scale and single occurrence of disturbance, which is unlikely to reflect grazing patterns in nature and more work is needed to upscale these predictions to larger disturbances with confidence.

Increased grazing pressure can occur as a result of increased herbivore populations (Choney et al., 2014; Kollars et al., 2017; Fourqurean et al., 2019; Buckee et al., 2021), and is already occurring as a result of climate change (Traill et al., 2009). Examples of this include the extension of herbivore distribution ranges due to tropicalisation (Hyndes et al., 2016) or concentration of populations in remaining suitable habitat in drying climates (Chambers, 2008). Meadows lacking an ability to rapidly recover can remain vulnerable if grazing continues or if other disturbances prevail (Dos Santos et al., 2013; O’Brien et al., 2018; Gangal et al., 2021). This can lead to overgrazing and, in extreme cases, the functional extinction of seagrass (Gangal et al., 2021). We have applied our findings to assess the risk of overgrazing of seagrass in this estuarine ecosystem, combining our new understanding of recovery timeframes, together with derived estimates of herbivore density, grazing pressure, and seagrass abundance across the estuary. Dos Santos et al. (2012) estimated a seagrass consumption rate of 394 g DW swan^–1^ day^–1^. Assuming an estimated swan population of ∼300 individuals (M. Bamford, personal communication) and a total biomass of seagrass in the Swan-Canning Estuary of 6 × 10^9^ g DW (∼150 g DW m^–2^ multiplied by 4.03 million m^2^ of seagrass; Kilminster and Forbes, 2014), then swans would consume 9% of seagrass biomass annually, assuming no recovery growth of the seagrass during that time. Thus, if the population of swans were to more than triple in the estuary, it could take more than 4 years for complete removal of seagrass from the system, in the absence of any recovery (Supplementary Table 8). The relatively low abundance of herbivores imposing comparatively low grazing pressure, and the rapid recovery of this seagrass suggests that these meadows are resilient to grazing now and into the future. This is unlikely to be the case for other ecosystems with high and/or dense herbivore populations and slower-growing seagrass species (Buckee et al., 2021; Gangal et al., 2021).

The response of seagrass cover and other meadow attributes that we observed in our study indicate the importance of choosing appropriate seagrass variables as indicators of condition. We defined RT in this study based on the aboveground cover of seagrass, but this did not indicate recovery of other plant attributes or meadow characteristics, despite a significant correlation between seagrass cover and these characteristics. This was evident in the leaf density and belowground biomass only having recovered to the condition of undisturbed seagrass in the least intense grazing treatment, despite our cover-based RT estimates projecting nearly all treatments at all locations to have recovered by the time the samples were collected. In this example, aboveground cover underestimated the RT of other seagrass attributes and is unlikely to indicate recovery of ecosystem services provided by seagrasses, such as habitat availability, nutrient cycling, and sediment stabilization (Hemminga and Duarte, 2000; Unsworth et al., 2015). Tiered approaches to monitoring that have clear recovery criteria and that incorporate indicators of recovery at several scales can provide efficient means of detecting and predicting trends in seagrass ecosystems and the services they provide (Neckles et al., 2012). While aboveground cover may underestimate the RT of other seagrass attributes, it can be beneficial as an observational measure of recovery as it is non-destructive.

## Conclusion

Aboveground cover of *H. ovalis* recovers within 3 months in the peak growth period from a single grazing event which removed up to 1 m^2^ of meadow. Increased extent of swan grazing not only lengthened the time for seagrass to recover, but also increased the variation in RT among different locations in one ecosystem. This suggests that some meadows are more resilient to grazing than others. Overall, seagrass recovery occurred consistently through vegetative growth from intact surrounding meadows, indicating the importance of maintaining healthy seagrass populations to facilitate recovery, though it is possible that at other times of year recovery from seed banks may be important. While recovery of aboveground cover was rapid (weeks to months), it may not accurately predict other seagrass attributes. A combination of recovery indicators can be considered in monitoring programs which aim to track recovery from disturbance. Multiple indicators of seagrass recovery should be incorporated into programs to increase confidence in the conclusion made regarding meadow regeneration. Failure to do so may limit the detection and predictability of seagrass recovery and put these precious ecosystems at risk.

## Data availability statement

The datasets generated for this study can be found in the Research Online repository at Edith Cowan University, available at: dx.doi.org/10.25958/fq3d-m857.

## Ethics statement

The animal study was reviewed and approved by Ethics Committee, Edith Cowan University (Project 21327).

## Author contributions

CO’D: conceptualization, methodology, formal analysis, investigation, data curation, writing – original draft preparation, visualization, project administration, and funding acquisition. PL and KM: conceptualization, methodology, writing – review and editing, and supervision. CW: formal analysis, investigation, data curation, writing – review and editing, and visualization. All authors contributed to the article and approved the submitted version.
