# Supplementary material for: Increased extent of waterfowl grazing lengthens the recovery time of a colonizing seagrass (Halophila ovalis) with implications for seagrass resilience
Source: Front Plant Sci. 2022 Aug 29;13:947109. doi: 10.3389/fpls.2022.947109 (PMC9465301; doi:10.3389/fpls.2022.947109)
Supplement: Supplementary file 1 [file Data_Sheet_1.pdf]

Supplementary Material

1 Supplementary Tables

Supplementary Table 1
Results of PERMANOVA testing for a) block and grazing intensity treatments (25, 50, 75 and 100% removal) nested within block on recovery time (RT) of *Halophila ovalis* based on aboveground cover (%) at four replicate locations across the Swan-Canning Estuary; b) block, grazing intensity treatments and the interaction on the meadow characteristics (leaf density, aboveground biomass and belowground biomass) 84 days following grazing simulation; and c) block, grazing intensity treatments and the interaction on the recovery mechanism variables 84 days following grazing simulation. Significant *p*-values ($p \leq 0.05$) in bold.

Source	df	F-value	Unique perms	*p*
**a) Recovery time**				
Block	3	2.973	9956	0.080
Treatment (Block)	3	12.489	9962	**0.001**
**b) Meadow characteristics**				
**Leaf density**				
Block	3	58.391	9953	**<0.001**
Treatment	5	10.819	9958	**0.001**
Interaction (Block x Treatment)	14	0.886	9906	0.566
**Aboveground biomass**				
Block	3	73.003	9957	**<0.001**
Treatment	5	6.473	9950	**0.004**
Interaction (Block x Treatment)	14	1.048	9916	0.420
**Belowground biomass**				
Block	3	8.142	9961	**<0.001**
Treatment	5	15.996	9950	**<0.001**
Interaction (Block x Treatment)	14	1.733	9947	0.082
**c) Recovery mechanisms**				
**Node density**				
Block	3	24.449	9940	**<0.001**
Treatment	5	31.441	9962	**<0.001**
Interaction (Block x Treatment)	14	1.369	9927	0.201
**Branching frequency (expressed as a proportion of node density)**				
Block	3	0.312	9958	0.822
Treatment	5	11.941	9952	**<0.001**
Interaction (Block x Treatment)	14	0.447	9921	0.952

**Branching potential frequency (expressed as a proportion of node density)**				
Block	3	8.084	9954	**0.001**
Treatment	5	3.872	9945	[a]**0.026**
Interaction (Block x Treatment)	14	1.424	9927	0.169
**Above to belowground biomass ratio**				
Block	3	11.269	9955	**<0.001**
Treatment	5	9.693	9949	**0.001**
Interaction (Block x Treatment)	14	0.663	9932	0.802
**Male reproduction frequency**				
Block	2	96.995	9955	**<0.001**
Treatment	5	2.295	9950	0.121
Interaction (Block x Treatment)	10	2.962	9936	**0.009**
**Female reproduction frequency**				
Block	1	2.603	9838	0.126
Treatment	5	1.187	9808	0.437
Interaction (Block x Treatment)	4	2.056	9948	0.115

Bold indicates significant p-values at $p \leq 0.05$.

[a] Pairwise post-hoc analysis indicated that significant differences only between the PControl and some treatments, but no difference between the PControl and Control, among treatments nor compared with the Control.

df = degrees of freedom; F values = pseudo-F by permutation; and *p*-values based on 9999 permutations

Supplementary Table 2

Mean cover of seagrass in Control and PControl plots at each replicate block over the duration of the experiment with Sign test paired t-test results for exact sig. (2-tailed) (significant *p*-values in bold where $p \leq 0.05$).

Block	Mean Control cover (%)	Mean PControl cover (%)	*p* (Exact Sign test; 2-tailed)
Lucky Bay	81.0	72.1	**0.063**
Pelican Point	67.2	61.7	0.375
Point Resolution	40.6	52.7	0.219
Rocky Bay	70.9	65.8	1.000

Supplementary Table 3

Minimum, median, and maximum swan counts recorded within line of sight by visual assessment at each replicate block location on the 8 sampling occasions during the experiment.

	Location			
Number of swans	Lucky Bay (LUB)	Pelican Point (PPT)	Point Resolution (PRS)	Rocky Bay (RCK)
Minimum	0	0	0	0
Median	10	18	0	0
Maximum	> 60	34	0	2

Supplementary Table 4

Summary of pairwise post-hoc test outputs to determine significant effect of simulated grazing treatments on recovery time (significant *p*-values in bold; $p \leq 0.05$).

	Groups		Recovery time	
			t	*p*
Treatments	25%,	50%	2.259	0.089
		75%	3.151	**0.013**
		100%	4.428	**0.012**
	50%,	75%	2.73	**0.030**
		100%	3.730	**0.032**
	75%,	100%	3.238	**0.028**

Supplementary Table 5

Summary of pairwise post-hoc test outputs to determine significant effect of simulated grazing treatments and block on biomass characteristics: leaf density, aboveground biomass (g DW) and belowground biomass (g DW) (significant *p*-values in bold; p ≤ 0.05).

	Groups		Leaf density		Aboveground biomass		Belowground biomass	
			t	*p*	t	*p*	t	*p*
Treatments	PControl,	Control	0.253	0.708	0.101	0.911	0.608	0.582
		25%	0.041	0.970	0.651	0.570	2.521	0.117
		50%	2.136	0.119	1.909	0.161	3.819	**0.044**
		75%	3.780	0.053	4.179	**0.035**	4.808	**0.028**
		100%	4.580	**0.033**	3.374	**0.039**	5.898	**0.023**
	Control,	25%	0.166	0.878	0.014	0.988	1.862	0.170
		50%	4.833	**0.042**	1.877	0.164	5.229	**0.023**
		75%	5.624	**0.024**	3.946	**0.034**	10.906	**0.006**
		100%	5.368	**0.017**	3.937	**0.032**	10.096	**0.008**
	25%,	50%	4.931	0.062	2.293	0.189	1.293	0.345
		75%	5.082	0.072	3.022	0.124	1.438	0.288
		100%	4.279	0.063	2.725	0.149	1.772	0.246
	50%,	75%	4.643	**0.031**	5.513	**0.021**	2.178	0.122
		100%	3.634	**0.049**	2.557	0.097	2.494	0.077
	75%,	100%	2.345	0.108	0.881	0.426	2.699	0.069
Blocks	LUB,	PPT	8.707	**<0.001**	8.836	**<0.001**	0.941	0.359
		PRS	11.510	**<0.001**	14.752	**<0.001**	4.209	**<0.001**
		RCK	0.892	0.370	3.451	**0.002**	1.870	0.075
	PPT,	PRS	3.732	**0.001**	6.784	**<0.001**	2.374	**0.027**
		RCK	6.753	**<0.001**	4.161	**<0.001**	2.414	**0.023**
	PRS	RCK	8.654	**<0.001**	8.619	**<0.001**	3.872	**0.001**

Supplementary Table 6

Summary of pairwise post-hoc test outputs to determine significant effect of simulated grazing treatments and block on recovery mechanisms: node density, branching frequency, branching potential frequency, and above to below ground biomass ratio (significant *p*-values in bold; $p \leq 0.05$).

	Groups		Node density		Branching frequency		Branching potential frequency		Above to below ground biomass ratio	
			t	*p*	t	*p*	t	*p*	t	*p*
Treatments	PControl,	Control	0.878	0.418	1.279	0.280	0.950	0.402	0.055	0.960
		25%	3.338	0.110	3.088	0.099	2.649	0.153	5.344	0.051
		50%	5.877	**0.016**	2.874	0.081	5.742	**0.021**	3.454	**0.036**
		75%	8.011	**0.008**	7.861	**0.009**	4.537	**0.012**	2.818	0.053
		100%	11.334	**0.004**	6.187	**0.007**	1.484	0.238	4.658	**0.027**
	Control,	25%	5.474	**0.037**	10.126	**0.010**	3.067	**0.035**	2.264	0.177
		50%	7.074	**0.001**	5.100	**0.015**	3.018	**0.012**	3.310	**0.039**
		75%	7.633	**0.001**	16.005	**0.002**	1.470	0.059	7.060	**0.018**
		100%	6.400	**0.001**	4.574	**0.025**	0.892	0.472	10.631	**0.004**
	25%,	50%	3.032	0.110	1.323	0.293	1.198	0.360	1.402	0.357
		75%	2.046	0.190	14.722	**0.018**	1.481	0.272	0.762	0.543
		100%	2.374	0.161	1.145	0.349	1.397	0.297	0.861	0.464
	50%,	75%	1.627	0.200	1.694	0.187	2.239	0.128	0.183	0.952
		100%	2.579	0.074	1.641	0.206	0.808	0.478	2.357	0.062
	75%,	100%	2.805	0.091	0.485	0.637	0.001	1.000	5.630	**0.011**
Blocks	LUB,	PPT	4.254	**<0.001**	NA	NA	4.447	**<0.001**	4.083	**<0.001**
		PRS	7.961	**<0.001**	NA	NA	2.612	**0.016**	6.125	**<0.001**
		RCK	2.808	**0.009**	NA	NA	3.103	**0.005**	3.507	**0.002**
	PPT,	PRS	2.696	**0.015**	NA	NA	2.191	**0.038**	2.120	**0.045**
		RCK	4.919	**<0.001**	NA	NA	1.157	0.264	0.148	0.887
	PRS,	RCK	5.969	**<0.001**	NA	NA	0.072	0.946	2.596	**0.018**

Supplementary Table 7

Summary of pairwise post-hoc test outputs to determine significant effect of the interaction between simulated grazing treatments and block on male reproduction frequency (significant *p*-values (Monte Carlo) in bold; $p \leq 0.05$).

	Block		LUB		PPT		PRS	
	Groups		t	*p*	t	*p*	t	*p*
Treatments	PControl,	Control	2.945	**0.042**	0.974	0.383	0.167	0.871
		25%	2.137	0.102	1.507	0.204	1.648	0.178
		50%	7.179	**0.003**	0.553	0.614	1.351	0.254
		75%	2.117	0.097	0.654	0.554	1.758	0.150
		100%	9.638	**0.001**	0.493	0.654	1.188	0.297
	Control,	25%	3.483	**0.026**	0.980	0.377	2.746	**0.049**
		50%	8.345	**0.001**	0.192	0.859	1.403	0.232
		75%	2.904	**0.044**	0.018	0.985	1.862	0.133
		100%	10.330	**0.001**	0.122	0.905	1.283	0.274
	25%,	50%	1.853	0.135	0.940	0.401	1.682	0.170
		75%	0.874	0.424	0.777	0.485	2.302	0.084
		100%	2.737	0.051	0.801	0.469	1.746	0.155
	50%,	75%	0.102	0.928	0.127	0.907	0.156	0.886
		100%	1.305	0.274	0.026	0.982	0.526	0.624
	75%,	100%	0.567	0.602	0.086	0.934	0.483	0.658

	Treatment		PControl		Control		25%		50%		75%		100%	
	Groups		t	p-value	t	p-value	t	p-value	t	p-value	t	p-value	t	p-value
Block	LUB,	PPT	12.392	**<0.001**	4.977	**0.006**	5.788	**0.006**	14.539	**<0.001**	4.226	**0.015**	16.773	**<0.001**
		PRS	8.458	**0.001**	4.665	**0.011**	6.825	**0.002**	2.661	**0.055**	2.454	0.069	5.861	**0.004**
	PPT,	PRS	0.755	0.493	0.120	0.911	2.916	**0.044**	1.439	0.221	1.870	0.136	1.314	0.264

Supplementary Table 8

Case study example of swan grazing pressure in the Swan-Canning Estuary: (1) Mean consumption rate of black swans in New Zealand (Dos Santos et al, 2012), (2) swan population (M. Bamford, pers. comms.) and seagrass habitat in the Swan-Canning Estuary (Kilminster & Forbes, 2014), and (3) estimated consumption of seagrass.

Grazing pressure			Seagrass abundance			
Swan abundance (ind.)	Consumption per swan (g DW d^{-1} ind^{-1})	Total consumption (kg d^{-1})	Area (m^2)	Biomass (g DW m^{-2})	Total biomass (kg DW)	Proportion of total biomass consumed (% d^{-1}) (% y^{-1})
~ 300 [a]	394 [b]	118.5	4×10^6 [c]	150 [c]	60,400	0.00024 (9)

[a] M. Bamford, pers. comms

[b] Dos Santos et al, 2012

[c] Kilminster & Forbes, 2014

2 Supplementary Figures

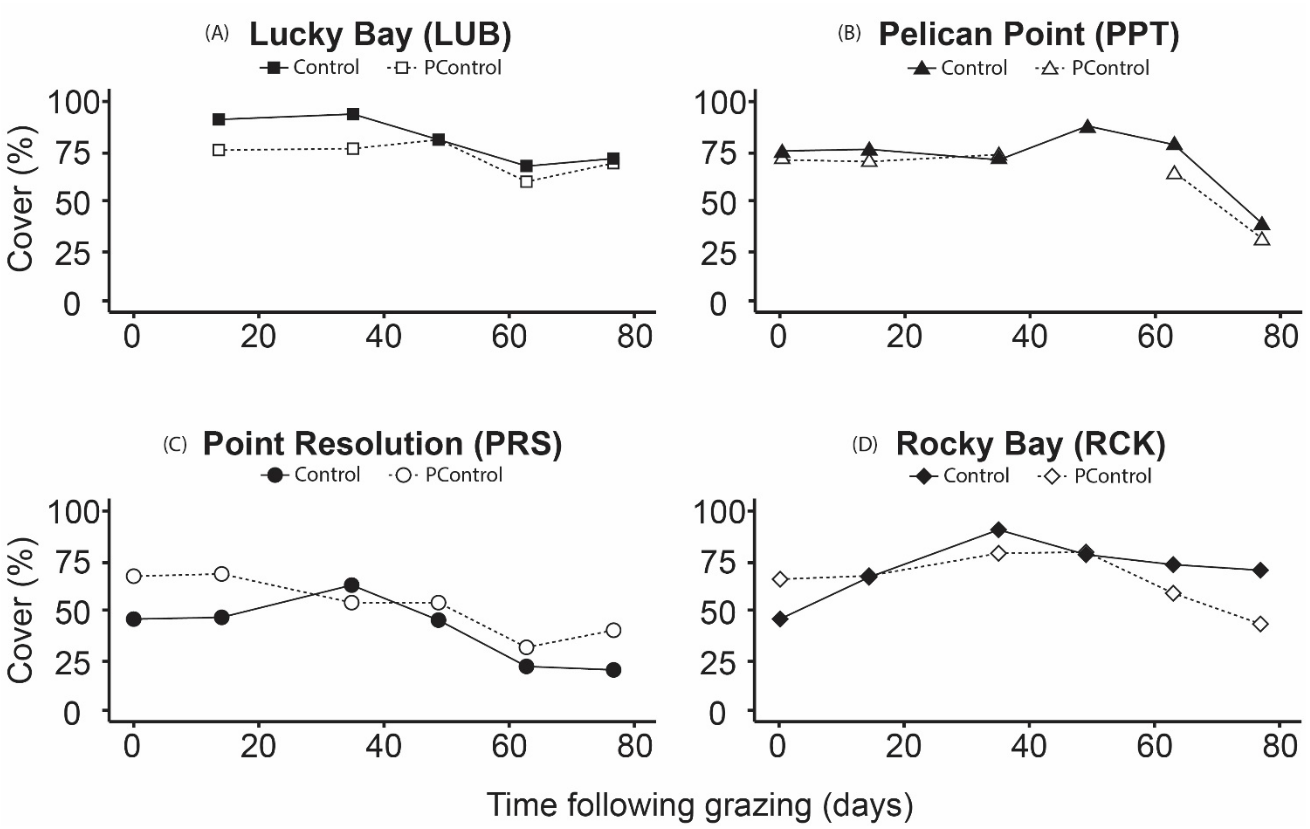

Supplementary Figure 1
Seagrass aboveground cover (%) in each of the Control (black with solid line) and PControl (white with dotted line) over time (d) at each of the replicate blocks in the Swan-Canning Estuary: Lucky Bay (LUB), Pelican Point (PPT), Point Resolution (PRS), and Rocky Bay (RCK). This shows seasonal declines in cover towards the end of the experiment (indicated by reduction in cover in both Control and PContol plots) and Table S2 shows the paired t-test significance. No data point indicates when data is not available.

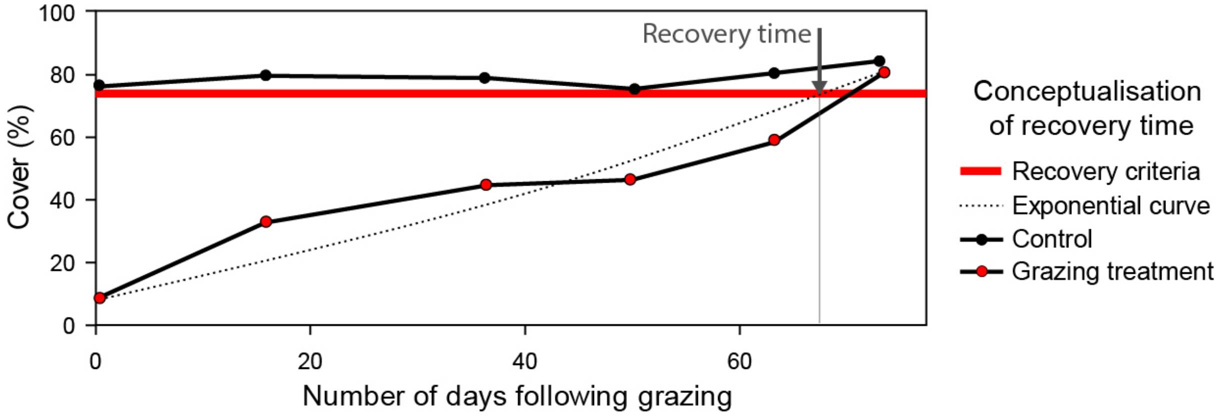

Supplementary Figure 2

Conceptualisation of recovery time calculations for seagrass aboveground cover (%) following simulated grazing (simulated on Day 0) over time (days). An example is shown for the cover of a treatment plot (black solid lines with white symbol) with an exponential curve fitting (dotted black line), which was used in calculating the minimum recovery time for seagrass cover, based on the recovery criteria (minimum cover of the Control plot – solid grey line). The exponential curve fitting reaching the criteria indicates when the cover of a treatment plot was extrapolated to estimate recovery time.

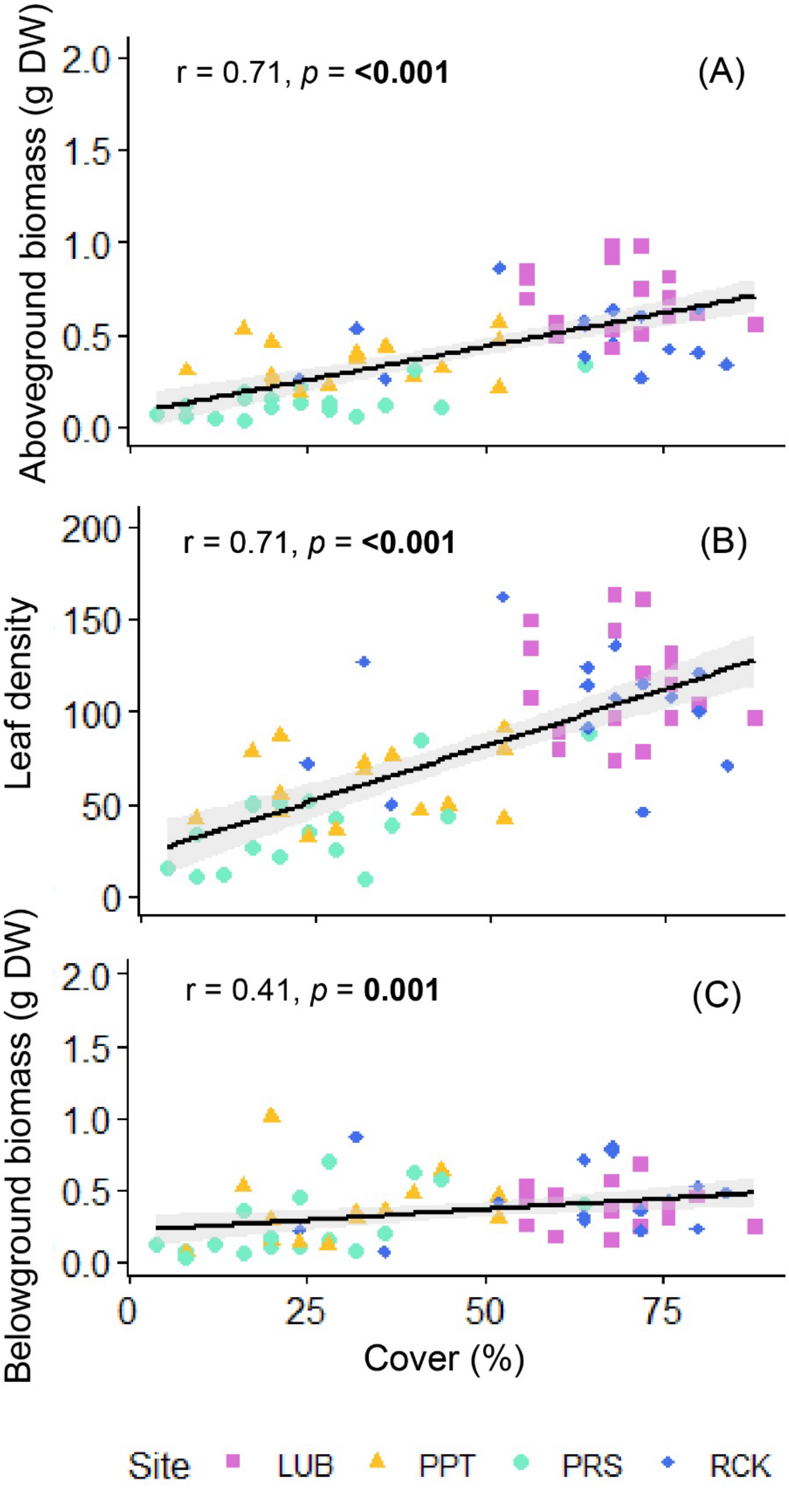

Supplementary Figure 3

Significant and moderate positive correlations between the leaf density and aboveground biomass compared to cover (%) and significant and slight positive correlation between the belowground biomass and cover (%), based on Spearman's rho correlation coefficient (r) and significant *p*-values in bold *(p* ≤ 0.05).
